# Supplementary figures and images for: Conservation and Targets of miR-71: A Systematic Review and Meta-Analysis
Source: Noncoding RNA. 2023 Jul 26;9(4):41. doi: 10.3390/ncrna9040041 (PMC10458147; doi:10.3390/ncrna9040041)

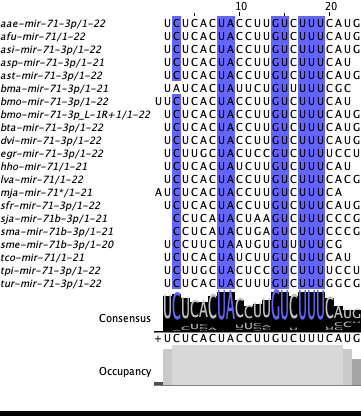

Supplement: Supplementary file 1 [file ncrna-09-00041-s001.zip › SupplementaryFigureS1.tiff]
